# Genetic and nutrient modulation of acetyl-CoA levels in *Synechocystis* for *n*-butanol production

**DOI:** 10.1186/s12934-015-0355-9

**Published:** 2015-10-16

**Authors:** Josefine Anfelt, Danuta Kaczmarzyk, Kiyan Shabestary, Björn Renberg, Johan Rockberg, Jens Nielsen, Mathias Uhlén, Elton P. Hudson

**Affiliations:** School of Biotechnology, KTH, Royal Institute of Technology, Science for Life Laboratory, Stockholm, Sweden; Novo Nordisk Foundation Center for Biosustainability, Technical University of Denmark, Hørsholm, Denmark; Department of Chemical and Biological Engineering, Chalmers Institute of Technology, Gothenburg, Sweden

**Keywords:** Cyanobacteria, Butanol, Biofuel, Metabolic engineering, Phosphoketolase, Starvation

## Abstract

**Background:**

There is a strong interest in using photosynthetic cyanobacteria as production hosts for biofuels and chemicals. Recent work has shown the benefit of pathway engineering, enzyme tolerance, and co-factor usage for improving yields of fermentation products.

**Results:**

An *n*-butanol pathway was inserted into a *Synechocystis* mutant deficient in polyhydroxybutyrate synthesis. We found that nitrogen starvation increased specific butanol productivity up to threefold, but cessation of cell growth limited total *n*-butanol titers. Metabolite profiling showed that acetyl-CoA increased twofold during nitrogen starvation. Introduction of a phosphoketolase increased acetyl-CoA levels sixfold at nitrogen replete conditions and increased butanol titers from 22 to 37 mg/L at day 8. Flux balance analysis of photoautotrophic metabolism showed that a Calvin–Benson–Bassham-Phosphoketolase pathway had higher theoretical butanol productivity than CBB-Embden–Meyerhof–Parnas and a reduced butanol ATP demand.

**Conclusion:**

These results demonstrate that phosphoketolase overexpression and modulation of nitrogen levels are two attractive routes toward increased production of acetyl-CoA derived products in cyanobacteria and could be implemented with complementary metabolic engineering strategies.

**Electronic supplementary material:**

The online version of this article (doi:10.1186/s12934-015-0355-9) contains supplementary material, which is available to authorized users.

## Background

Photosynthetic microbes are promising chemical and biofuel hosts as they circumvent the use of plant-based feedstocks and thus eliminate a large cost for current-generation biofuels. Cyanobacteria in particular are attractive due to extensive genetic and biochemical characterization, ease of genetic modification, and diverse ecology and habitat. Model cyanobacteria such as *Synechocystis* sp. PCC 6803 and *Synechococcus* spp. have been modified with heterologous biofuel pathways to produce biofuels and biofuel precursors such as alcohols and free fatty acids [1, 2]. Recent work has aimed to increase metabolic flux toward biofuel production in cyanobacteria through pathway engineering [3, 4], enzyme stability and selectivity [5], and anoxic culturing [6, 7]. From these reports, as well as experimental and in silico metabolic flux analyses, it is clear that photoautotrophic biofuel production can be limited by carbon flux through acetyl-CoA, which is significantly lower than during heterotrophic growth [8]. For heterologous fermentative pathways, co-factor choice is also an important concern due to a prevalence of NADPH in cyanobacteria from the light reactions.

Nitrogen content of the culture media can induce drastic changes to the central carbon metabolism of non-diazotrophic cyanobacteria such as *Synechocystis*. Under nitrogen deplete conditions, carbon fixation and de novo metabolite synthesis does occur [9], although at a lower rate. Furthermore, carbon is directed to storage compounds such as glycogen and polyhydroxybutyrate (PHB) [10], and the redox state of the cell is altered as NADPH accumulates [11]. The concerted redirection of carbon throughout central metabolism could be exploited for biofuel production.

Here, we incorporated a fermentative butanol pathway into a *Synechocystis* sp. PCC 6803 mutant that is impaired in PHB synthesis. Similar to PHB accumulation in wild-type *Synechocystis*, we found that butanol specific titers (butanol produced per cell) increased with lower nitrogen or phosphorus levels in the starting culture. By investigating the metabolic changes at nitrogen starvation, we subsequently identified potential driving forces and limitations for butanol production at nitrogen depletion. A phosphoketolase for increased acetyl-CoA was introduced in order to partly simulate starvation conditions. The results show the utility of phosphoketolase in re-directing autotrophic metabolism, which could be used in addition to other metabolic engineering strategies.

## Results and discussion

### Expression of butanol-production pathway in nitrogen replete and deplete conditions

The *n*-butanol fermentation pathway of *Clostridia* proceeds from acetyl-CoA in six steps and has been adapted to other bacterial hosts [12, 13] as well as yeast [14, 15]. A chimeric version of this pathway has been developed which replaced the *Clostridia* thiolase, acetoacetyl-CoA reductase, enoyl-CoA hydratase, and enoyl-CoA reductase enzymes with more robust variants [16]. This chimeric pathway supports higher flux than the *Clostridia* pathway when expressed in *E. coli* and includes an irreversible reduction of crotonyl-CoA by Ter.

**Fig. 1:**
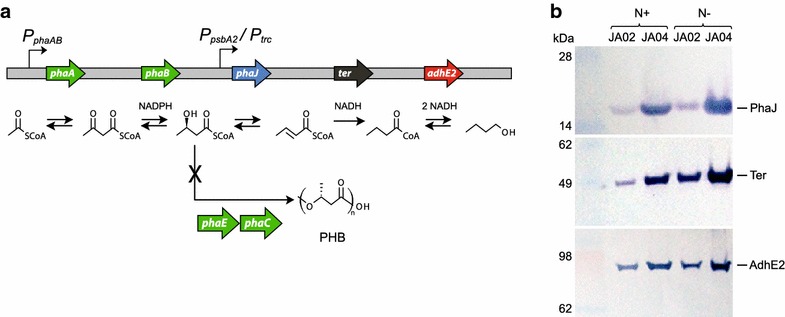
Expression of *n*-butanol biosynthesis genes. **a** Scheme of a constructed *n*-butanol biosynthesis route in *Synechocystis*, with a competing PHB synthesis pathway deleted. *Green arrows* represent native genes, while *blue*, *black* and *red arrows* represent heterologously expressed genes. **b** Western blot of butanol biosynthesis enzymes from strain JA02 (*phaJ ter adhE2* under *P*_*psbA2*_) and JA04 (*phaJ ter adhE2* under *P*_*trc*_) at nitrogen replete (N+) and deplete (N−) conditions. 12 μg total protein was loaded from each sample

To produce *n*-butanol in *Synechocystis*, we made use of a native β-ketothiolase (*phaA*) and acetoacetyl-CoA reductase (*phaB*), which are involved in PHB production [17]. The terminal three enzymes were from the chimeric *n*-butanol pathway (*phaJ*, *ter*, *adhE2*) and were expressed from a low-copy, replicative plasmid under the moderate-strength *Synechocystis* promoter *P*_*psbA2*_ or the strong promoter *P*_*trc*_. Production of PHB was eliminated by genomic deletion of the PHB synthase genes *phaE* and *phaC*, resulting in strain JA02 (Fig. 1a; Table 1). Expression of the heterologous enzymes was determined by Western blot detection of N-terminal immunochemical tags and by RT-qPCR (Fig. 1b; Additional file 1: Fig. S2).

**Table 1:** *Synechocystis* strains used in this study

Strain	Plasmid	Genome modification	Notes
JA01	pJA2-*P*_*psbA2*_ *phaJ ter adhE2*	None	Butanol biosynthesis genes on replicating plasmid
JA02	pJA2-*P*_*psbA2*_ *phaJ ter adhE2*	Δ*phaEC*::SpR	PHB synthase deletion eliminates PHB production
JA03	pJA2-*P*_*psbA2*_ *phaJ ter adhE2*	Δ*phaEC*::SpR, ΔNSI::*P*_*trc*_ *phaAB* CmR	Overexpression of the native *phaA* and *phaB genes*
JA04	pJA8-*P*_*trc*_ *phaJ ter adhE2*	Δ*phaEC*::SpR	*P*_*psbA2*_ replaced with the stronger trc promoter
JA05	pJA8-*P*_*trc*_ *phaJ ter adhE2*	Δ*phaEC*::SpR, ΔNSI::*P*_*trc*_ *phaAB* CmR	
JA06	None	ΔNSI::*P*_*trc*_ *xfpk* CmR	Expression of a phosphoketolase
JA07	pJA8-*P*_*trc*_ *phaJ ter adhE2*	Δ*phaEC*::SpR ΔNSI::*P*_*trc*_ *xfpk* CmR	

After 3 days of nitrogen deplete conditions, transcript levels of *phaA* and *phaB* increased approximately nine and eightfold, respectively, while *phaJ*, *ter*, and *adhE2* transcripts changed less than twofold. The upregulation of *phaA* and *phaB* during nitrogen starvation is consistent with an activation of the PHB pathway. The relative amounts of heterologous PhaJ, Ter and AdhE2 proteins increased after 3 days of nitrogen depletion (Fig. 1b). This apparent increase is likely due to degradation of phycobilisomes and overall reduction in protein biosynthesis during starvation [18]. Loss of phycobilisomes increases the fraction of total protein of the heterologous enzymes. Notably, an extended culturing period of strain JA04 in nitrogen-deplete conditions showed a high and stable expression of Ter after 12 days of nitrogen starvation (Additional file 1: Fig. S2), indicating that heterologous protein degradation is not a factor during the starvation durations used here. Cell growth was restored after 2 weeks of starvation by inoculation into fresh BG-11 medium.

### Production of butanol under nitrogen replete and deplete conditions

**Fig. 2:**
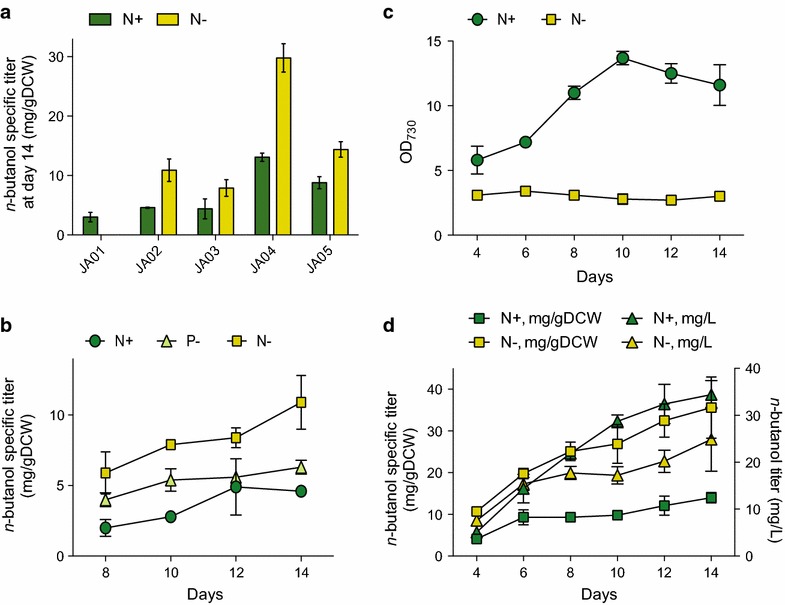
Butanol production and growth curves at nitrogen replete (N+), nitrogen deplete (N−) and phosphorous deplete (P−) conditions. **a** Butanol accumulation after 14 days. Butanol production was abolished during nitrogen starvation if PHB synthase was present (JA01). Deletion of PHB synthase (JA02) resulted in increased butanol production during starvation. Enhanced expression of PhaJ, Ter and AdhE2 (JA04) increased butanol accumulation threefold. **b** Time course of butanol accumulation from strain JA02. Specific production of butanol increased up to twofold (P−) or threefold (N−). **c** Optical density in strain JA04 cultures. Cell densities remain constant during extended culturing in N− media. **d** Butanol titers and specific titers over time from strain JA04. The average specific productivities during day 4–14 of cultivation were 1.1 mg/gDCW/day (N+) and 2.7 mg/gDCW/day (N−). *Error bars* represent SD of biological triplicates

We observed butanol accumulation in the culture medium from strains JA01 and JA02, which contained the heterologous butanol-biosynthesis genes under *P*_*psbA2*_ on a replicating plasmid (Fig. 2a). Butanol accumulated to 6 mg/L from JA01, containing PHB synthase genes, and 12 mg/L for JA02 after 14 days in nitrogen-replete conditions (Additional file 1: Fig. S3). Under nitrogen starvation, strain JA01 did not produce butanol, presumably due to diversion of the 3HB-CoA intermediate to PHB, and was therefore excluded from further study. Butanol titers from JA02 were reduced (7 mg/L for samples starved for 14 days) during nitrogen starvation, due to reduction of cell growth. However, specific titers of butanol (mg *n*-butanol/gDCW) increased up to threefold (Fig. 2b). Phosphorous limitation increased specific titers twofold, consistent with a previous report showing increased 3-HB production by *Synechocystis* during phosphorous starvation [19].

The specific titer of butanol from JA02 after a 7 days batch culture was inversely proportional to the starting nitrate content in the medium (Additional file 1: Fig. S3). A similar trend was observed for PHB accumulation in wild-type *Synechocystis*, where PHB began to accumulate only after 3 days of cultivation, when an estimated 50 % of the nitrate in the media had been depleted. The sensitivity of butanol production to nitrate levels is indicative of the metabolic re-routing that occurs during nitrogen starvation and suggests that it may be possible to optimally balance cell biomass and biofuel production by manipulation of nitrogen conditions in continuous culture.

**Fig. 3:**
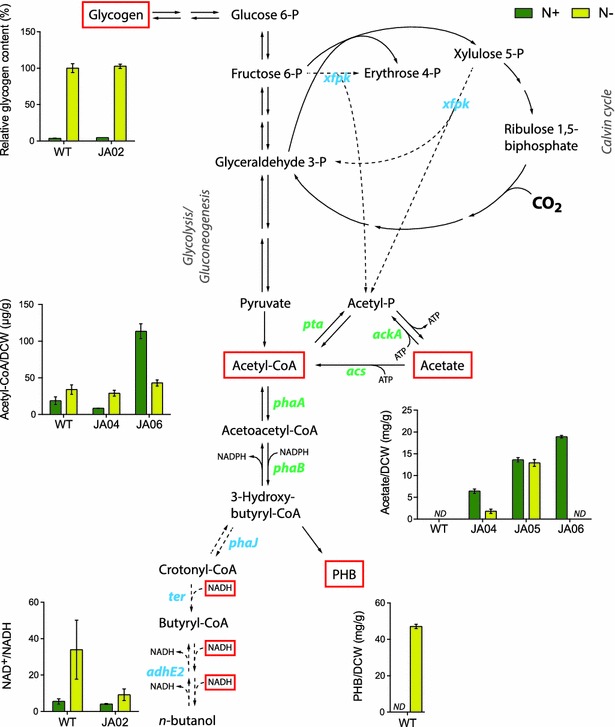
Overview of metabolic effects on *Synechocystis* during nitrogen starvation. Metabolites (*red squares*) with potential influence on butanol synthesis were quantified at nitrogen replete (N+) and deplete (N−) conditions. Glycogen levels are relative to wild type at N−, which is defined as 100 %. Heterologous enzymes (in *blue*): *xfpk* (phosphoketolase, *B. breve*), *phaJ* (enoyl-CoA hydratase, *A. caviae*), *ter* (trans-enoyl-CoA reductase, *T. denticola*) and *adhE2* (bifunctional aldehyde/alcohol dehydrogenase, *C. acetobutylicum*). Native enzymes (in green): *phaA* (beta-ketothiolase), *phaB* (acetoacetyl-CoA reductase), *pta* (phosphotransacetylase), *ackA* (acetate kinase), *acs* (acetyl-CoA synthetase). *Error bars* represent SD of biological duplicates for the acetyl-CoA, acetate and NAD^+^/NADH quantifications and biological triplicates for the glycogen and PHB quantifications. *ND* not detectable. Absolute metabolite values are listed in Additional file 1: Table S1

In order to identify bottlenecks in the butanol synthesis pathway, we modulated expression of the *phaAB* operon and the *phaJ ter adhE2* operon separately. In strain JA04, *phaJ ter adhE2* was placed under the strong promoter *P*_*trc*_. Western blot analysis confirmed a significant increase in expression of all three enzymes compared to strain JA02, and butanol specific titers increased threefold both at nitrogen replete and deplete conditions (Fig. 2a). Total butanol titers were 34 and 25 mg/L after 14 days, with 10-day average specific productivities of 1.1 and 2.7 mg/gDCW/day at nitrogen replete and deplete conditions, respectively. Though cell growth ceased during nitrogen starvation, butanol continued to accumulate in the culture medium for 14 days. Interestingly, overexpression of *phaAB* in the background of strains JA02 and JA04 (creating JA03 and JA05, respectively) *decreased* butanol titers. Protein levels of PhaA and PhaB in these strains were high, and confirmed by Western blot (Additional file 1: Fig. S4). The decreased butanol production of strain JA05 was accompanied by increased acetate secretion, and this was most pronounced in nitrogen deplete conditions (Fig. 3). Acetate formation from acetyl-CoA could be catalyzed by Pta-AckA. Transcriptomics data showed that during nitrogen depletion phosphotransacetylase (*pta*) was upregulated and acetyl-CoA synthetase (*acs*) and ATP synthase were downregulated [20], which could reduce the ability to reassimilate secreted acetate. Overexpression of PhaAB is expected to increase the rates of both the forward and reverse reactions in the conversion of acetyl-CoA to 3HB-CoA. However, increased flux to 3HB-CoA was not directed toward butanol, due to inefficiencies in the downstream (PhaJ Ter AdhE2) reactions. Without an efficient kinetic trap for 3HB-CoA, re-oxidation and hydrolysis to acetate occurs.

The dual problems of unfavorable reaction thermodynamics and weak acetyl-CoA driving force in the PhaA condensation reaction were addressed by Lan and Liao in a *n*-butanol production system in *Synechococcus* PCC 7942 (*Synechococcus*). They implemented a decarboxylative condensation of malonyl-CoA and acetyl-CoA (catalyzed by NphT7) to form acetoacetyl-CoA and thus overcome the weak acetyl-CoA driving force. This altered pathway increased *n*-butanol titers fourfold relative to thiolase-catalyzed condensation [4]. Butanol titer from the NphT7-pathway in *Synechococcus* was 8 mg/L after 8 days, lower than the PhaA-pathway in *Synechocystis* reported here (22 mg/L after 8 days). The other enzymes in the butanol pathway are similar. A larger acetyl-CoA pool could contribute to a more efficient thiolase pathway in *Synechocystis* than in *Synechococcus*. We measured acetyl-CoA pools in both *Synechocystis* and *Synechococcus* during mid-log phase and found a fourfold larger pool in *Synechocystis* (Additional file 1: Fig. S5). This is comparable to a 6.5-fold difference previously reported [21], though absolute acetyl-CoA values are different, likely due to different metabolite extraction methods.

### Nitrogen starvation increases acetyl-CoA pools and oxidizes NADH pools

We next investigated the metabolic effects of nitrogen starvation in wild-type cultures by quantifying intracellular acetyl-CoA, NADH, glycogen and PHB under nitrogen-replete conditions and after 72 h of nitrogen starvation. Nitrogen deplete conditions increased the acetyl-CoA pool 2-threefold in wild-type and the butanol strain JA04 (Fig. 3). Increased acetyl-CoA thus correlates with increased specific butanol titers. The lower acetyl-CoA levels in JA04 relative to wild type may be due to acetate secretion from this strain. A recent study found that acetyl-CoA concentrations were increased with cultivation time as the phosphate and nitrate supplies were gradually consumed [19]. In a separate study, acetyl-CoA levels rose for 2 days after starvation and remained stable for the subsequent 3 days [10]. The increase in acetyl-CoA during nitrogen starvation can be beneficial for biosynthesis of other acetyl-CoA products [22]. An impaired ability to synthesize carbon storage polymers during starvation could potentially also increase the substrate availability for butanol production, as illustrated by the increased butanol titers from the PHB-deficient strain JA02. We note that glycogen accumulation however was unaffected in PHB-deficient mutants (Fig. 3), indicating that these two are not linked directly.

In addition to acetyl-CoA precursor, the abundance of redox cofactors could also influence butanol biosynthesis in *Synechocystis*. In cyanobacteria, the reported NADPH/NADH ratio ranges from 1.1 to 7 [21, 23, 24], significantly higher than the 0.31 of *E. coli* [25]. Consequently, the use of enzymes with NADPH preference in cyanobacteria pathways have been shown to increase production of ethanol (a NADPH-utilizing alcohol dehydrogenase) [7], butanol (a NADPH-utilizing butyryl-CoA reductase) [4] and lactate (a mutant lactate dehydrogenase which showed NADPH activity) [26]. During nitrogen starvation, the NADP(H) pool becomes more reduced; the NADPH/NADP^+^ ratio increased approximately threefold after 72 h of nitrogen starvation [11]. In contrast, we found that the NAD(H) pool becomes *oxidized* during nitrogen deplete conditions, as the NAD^+^/NADH ratio increased sixfold after nitrogen starvation for 72 h (Fig. 3). Deletion of PHB synthesis partially alleviated this effect; the NAD^+^/NADH ratio increased only twofold during nitrogen starvation in the PHB-negative strain JA02.

The chimeric butanol pathway used here requires at least three NADH (*ter*, *adhE2*) per butanol produced. The cofactor preference of *phaB* is presumed to be NADPH, but has not been determined. We also note that a native NADPH-specific *Synechocystis* alcohol dehydrogenase (AdhA, *slr1192*) is expressed and is active toward butyraldehyde and may therefore catalyze the reduction of butyraldehyde to butanol [27], thus changing the cofactor requirements. In either case, the depletion of NADH during nitrogen starvation, and the fact that deletion of PHB synthesis was necessary to form butanol, suggest that NADH supply may not be sufficient to allow for high butanol titers in *Synechocystis*. Introduction of a transhydrogenase could increase the NADH/NAD+ ratio and has been demonstrated in cyanobacteria for increased lactic acid synthesis [28]. However, the expression level of transhydrogenase must be carefully tuned to balance the redox need of its corresponding NADH-requiring pathway, as illustrated by the unstable strain resulting from transhydrogenase expression in wild-type.

### Phosphoketolase increases theoretical butanol production in the photoautotrophic condition

While nitrogen starvation of *Synechocystis* increases *specificn*-butanol productivity, total titers are reduced after approximately 6 days of cultivation due to a reduction of carbon fixation rate and cessation of cell growth. We sought a way to increase acetyl-CoA pools without these negative effects. Phosphoketolases catalyze the cleavage of xylulose 5-phosphate (Xu5P) or fructose 6-phosphate (F6P) to acetyl-P and glyceraldehyde-3-P or erythrose-4-P and, in combination with the phosphate acetyltransferase, are an alternative pathway from sugars to acetyl-CoA. Overexpression of a phosphoketolase in yeast improved free fatty acid production [29] and PHB accumulation [30]. A phosphoketolase was recently also used in a synthetic non-oxidative glycolysis pathway in *E. coli*, which resulted in complete conversion of F6P and Xu5P to acetyl-phosphate [31]. During growth on glucose, phosphoketolase cleavage of pentose-phosphate intermediates may “pull” flux into the pathway, resulting in increased levels of both NADPH and acetyl-CoA. However, NADPH negatively regulates the OPP pathway, so that the utility of a phosphoketolase could be lost at high NADPH levels. In cyanobacteria, where flux through the OPP is small under photoautotrophic conditions [32], a phosphoketolase could instead divert intermediates to acetyl-CoA from the Calvin-cycle, which is positively regulated by NADPH. The Xfpk reaction in *Synechocystis* (CBB-PKT pathway) allows formation of acetyl-CoA from two CO_2_ instead of the three required by the CBB-EMP pathway. Because the CBB-PKT pathway requires fewer Rubisco turnovers per formed acetyl-CoA, there is a kinetic and energetic benefit. Xfpk may therefore allow for higher butanol yields (per ATP) in a light-limiting, autotrophic condition. *Synechocystis* has two *putative* native phosphoketolases as predicted through sequence homologies to known phosphoketolases, but their activity has not been experimentally verified [32].

**Fig. 4:**
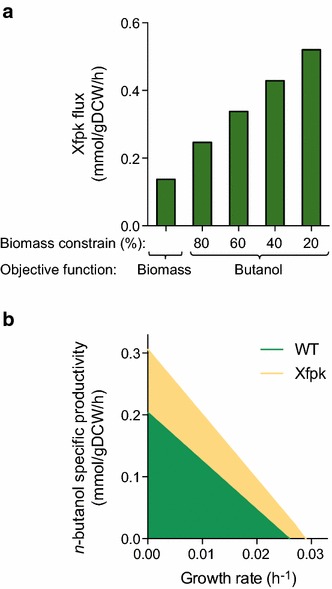
Effect of phosphoketolase on flux balance analysis solutions in *Synechocystis.* **a** Flux through Xfpk in FBA solutions at autotrophic conditions when butanol production is used as objective function and biomass is fixed as a percentage of μmax. The photon uptake was constrained to 18.7 mmol/gDCW/h. **b** Phenotypic phase plane for butanol and biomass in autotrophic conditions. Presence of Xfpk increased theoretical butanol productivity

We used flux balance analysis (FBA) to predict the effect of phosphoketolase reactions on *n*-butanol production in *Synechocystis* PCC 6803. We added the *n*-butanol reactions and the two Xfpk reactions to a genome scale model [33] and found FBA solutions for optimizing either biomass or *n*-butanol as objective functions in a light-limited autotrophic condition. When biomass was used as the objective function, the Xfpk reaction (either F6P or Xu5P cleavage) provided 40 % of all acetyl-CoA. The maximum growth rate also increased when Xfpk carried flux (Table 2). The Xfpk reactions thus increased the ATP-efficiency of biomass formation (more biomass formed per ATP generated). When butanol was set as the objective function, flux through Xfpk increased such that *all* acetyl-CoA was supplied through the CBB-PKT reactions and there was no flux through lower glycolysis (Fig. 4a). The CBB-PKT pathway is thus theoretically the most ATP-efficient route from CO_2_ to butanol. We next fixed biomass growth at a certain rate and optimized for butanol production to construct a phenotypic phase plane (PPP) with and without Xfpk present. The PPP showed increased butanol production at all combinations of biomass and butanol production (Fig. 4b). These results suggest that Xfpk allows more efficient use of light-derived ATP to produce both butanol and biomass. To estimate the ATP cost of butanol production, we summed all ATP consuming fluxes calculated by FBA when optimizing for butanol production. ATP costs of butanol (ATP used per butanol produced) decreased from 35.7 to 19.6 (45 %) when Xfpk was present.

**Table 2:** Fluxes obtained from *Synechocystis* autotrophic FBA solutions with and without Xfpk present when butanol or biomass production is the objective function Z

	Z = Butanol		Z = Biomass	
	Xfpk−	Xfpk+	Xfpk−	Xfpk+
BuOH (mmol/gDCW/h)	0.204	0.306	0	0
Biomass (1/h)	0	0	0.026	0.029
ATP (mmol/gDCW/h)	7.3	6.0	5.7	6.3
ATP/BuOH (mmol/mmol)	35.7	19.6	–	–

Photon uptake was constrained to 18.7 mmol/gDCW/h. Na+ and ATP-dependent CO_2_ uptake were allowed

### Phosphoketolase increases acetyl-CoA pools and *n*-butanol production

**Fig. 5:**
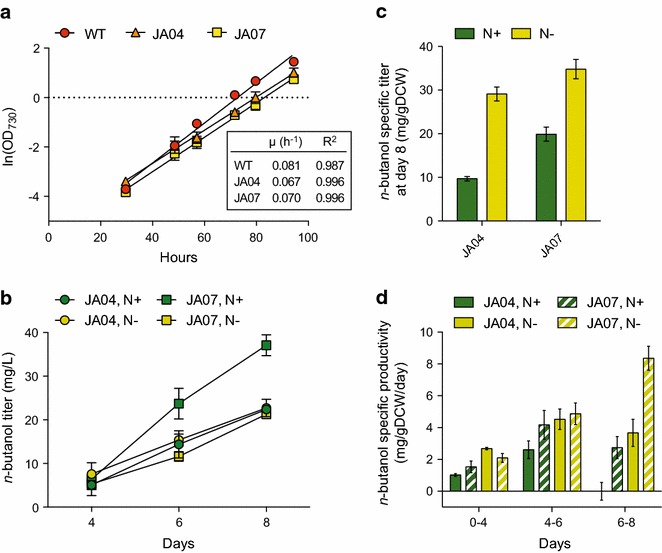
Butanol production and growth rate of Xpfk strain at nitrogen replete (N+) and nitrogen deplete (N−) conditions. **a** Growth of butanol producing strains with (JA07) and without (JA04) Xfpk overexpression at nitrogen replete conditions. Panel: Specific growth rates calculated from slopes. **b** Time course of butanol accumulation. Phosphoketolase expression increased titer 1.7-fold at 8 days. **c** Butanol accumulation after 8 days. The specific production of butanol increased twofold at N+ when expressing an exogenous phosphoketolase (JA07). **d** Specific productivity of butanol over time. Phosphoketolase significantly increase specific productivity at one week of cultivation. *Error bars* represent SD of biological triplicates

Based on the predicted effect of Xfpk, we hypothesized that butanol productivity in *Synechocystis* could be increased through overexpression of a phosphoketolase. No native phosphoketolase has so far been characterized in *Synechocystis*, although two putative phosphoketolases (*slr0453* and *sll0529*) have been identified through homology search [34]. Transcripts from both genes have also been detected in a previous study using RNA-Seq [20]. A phosphoketolase (*xfpk*) from *Bifidobacterium breve* was expressed under the control of *P*_*trc*_ in the NSI of strain JA04 and wild type, resulting in strain JA07 and JA06, respectively. We chose the *B. breve* Xfpk due to its activity towards both Xu5P (29 U/mg) and F6P (15 U/mg) and a solved crystal structure [35]. Expression of Xfpk in strain JA07 increased specific *n*-butanol titers twofold in nitrogen-replete conditions (Fig. 5b, c). Xfpk increased acetyl-CoA levels sixfold in strain JA06 compared to wild type under nitrogen-replete conditions (Fig. 3), and also led to acetate secretion, suggesting that acetyl-CoA formation could be enhanced further through deletion of acetate kinase. Interestingly, Xfpk had minimal effect on acetyl-CoA and butanol production during nitrogen starvation, despite high expression (Additional file 1: Fig. S6). This is unexpected, as the Xfpk substrate F6P is expected to be at high concentrations during the gluconeogenesis that occurs during nitrogen starvation.

Maximum specific butanol productivity was 8 mg/gDCW/day for strain JA07 under nitrogen depletion. This is approximately tenfold less than a recently reported *Synechococcusn*-butanol strain [5]. In that study, the oxygen-tolerant CoA-acylating propionaldehyde dehydrogenase (PduP) from *Salmonella enterica* was key for high butanol productivity. Considering the oxygen sensitivity of AdhE2 [36], it is possible that the higher specific productivities observed during nitrogen starvation are a result of the decreased oxygen formation from PSII in this condition [37]. We attempted to increase butanol titers by replacing AdhE2 in strain JA04 with either PduP or PduP-YqhD (alcohol dehydrogenase from *E. coli*). We did not see increased butanol titers at either nitrogen replete or deplete conditions in these strains (Additional file 1: Fig. S7), likely due to the low expression of PduP as measured by Western Blot. Further optimization of PduP expression is necessary to improve butanol titers in this strain.

## Conclusions

We examined the effect of nitrogen starvation on carbon distribution and butanol production in *Synechocystis* sp. PCC 6803. We found that elimination of PHB synthase allows butanol production under nitrogen deplete conditions, indicating a redirected carbon flux from PHB to butanol. Specific production of butanol was improved threefold by nitrogen starvation, and one of the main driving forces for this change was an increase in acetyl-CoA. However, low NADH levels may limit butanol production in this condition. Expression of a phosphoketolase increased both the titer and specific production of butanol (1.7- and 2-fold, respectively) at nitrogen replete conditions by effectively forming a shortcut from the Calvin cycle to acetyl-CoA production. The intracellular acetyl-CoA concentration was increased sixfold by the introduction of phosphoketolase, which can serve as a valuable strategy for other acetyl-CoA-derived products, including alternative, non-fermentative routes to butanol [36].

## Methods

### Strain cultivation

*Synechocystis* sp. PCC 6803 strains were cultivated in BG-11, nitrate depleted BG-11 (BG-11_0_) or phosphate depleted BG-11 (BG-11_p−_) medium supplemented with 50 mM NaHCO_3_ and buffered to pH 7.8 with 25 mM HEPES. Cultures were grown in a climatic chamber (Percival Climatics SE-1100) at 28 °C, 50 μE/s/m^2^ illumination, and CO_2_ at 1 % v/v. Cultures for butanol quantification were grown in 24-well plates sealed with parafilm in order to minimize butanol evaporation. The cultures were unsealed once per day and gently mixed through pipetting to allow for proper CO_2_ supply. Pre-cultures for butanol quantification were prepared by inoculation from a mature culture to OD_730_ 0.1. When OD_730_ reached 1, the cells were washed in BG-11, BG-11_0_ or BG-11_p−_ and resuspended in the same growth medium to OD_730_ 1. The cultures were prepared in triplicates and *n*-butanol was quantified after 4–14 days of growth. Butanol production dependence on nitrate concentration in the growth medium was measured from cultures inoculated to OD_730_ 1 in BG-11 with varying nitrate concentrations. Samples were prepared in triplicates and grown for 7 days at 100 % (1.5 g/L), 25, 5, 1 and 0 % of normal nitrate concentration in BG-11. Cultures for Western blotting and for glycogen, acetyl-CoA, acetate, PHB and NADH/NAD^+^ quantification were inoculated from a mature culture to OD_730_ 0.1 in BG-11 and grown until OD_730_ 1. Cells to be nitrate or phosphate depleted were then washed and resuspended in BG-11_0_ and BG-11_p−_, respectively to OD_730_ 1 and cultured for three additional days.

### Strain modification

Butanol producing *Synechocystis* strains were generated through introduction of the genes *phaJ* (enoyl-CoA hydratase from *A. caviae*), *ter* (trans-enoyl-CoA reductase from *T. denticola*) and *adhE2* (bifunctional aldehyde/alcohol dehydrogenase from *C. acetobutylicum*) on the replicating vector pJA2 [20]. pJA2 is a broad-host, low-copy vector expressing inserted genes under the promoter *P*_*psbA2*_ and is derived from vector pPMQAK1 constructed by Huang et al. [38]. Due to difficulties in restriction digest of this RSF1010 plasmid [39], we performed cloning using PCR-amplified vector. The pJA2 backbone was PCR amplified and digested with *Xba*I and *Pst*I restriction enzymes. Genes to be inserted into pJA2 were amplified from the start codon to the stop codon with gene-specific primers where an *Xba*I site was introduced in the 5′-overhang of the forward primer and a *Pst*I site in the overhang of the reverse primer. The inserts were *Xba*I/*Pst*I digested and ligated with the pJA2 backbone. The exogenous genes *phaJ*, *ter* and *adhE2* (Uniprot entry O32472, Q73Q47 and Q7DFN2, respectively) were codon optimized and synthesized as one single construct by Epoch Life Science. A unique tag enabling immunodetection was introduced directly downstream of the start codon in all three genes; c-Myc tag for *phaJ*, FLAG tag for *ter* and Strep-tag II for *adhE2*. Ribosome binding sites (BioBrick BBa_B0034, http://partsregistry.org) were introduced 6 bp upstream of the start codon of the second and third gene in the construct.

Plasmid pJA8 was constructed by replacing *P*_*psbA2*_ in pJA2 with *P*_*trc*_ followed by the BBa_B0034 ribosome binding site. *P*_*psbA2*_ was removed from pJA2 through digestion with *Asc*I and *Xba*I, and the trc promoter was amplified with primers designed to introduce the same restriction sites in the 5′ and 3′ end, respectively, of the promoter (for promoter sequences, see Additional file 1). PduP (CoA-acylating propionaldehyde dehydrogenases from *S. enterica*, Uniprot entry V2D4V9) was codon optimized and synthesized by Integrated DNA Technologies, with a c-Myc tag introduced N-terminally. YqhD (alcohol dehydrogenase from *E. coli*, A0A0E1M1H8) was PCR amplified from TOP10 cells with primers introducing an N-terminal Strep-tag II. These genes were inserted downstream of *ter*, replacing *adhE2*.

Replacement of *phaEC* was achieved via double homologous recombination insertion of a spectinomycin resistance cassette. Transformation plasmids contained 1000 bp homology flanking the antibiotic resistance cassette. All knockout plasmids were pUC19 variants and were designed using the online program Gibthon 2.0. Plasmids were constructed using 4 or 5-piece isothermal assembly [40] and confirmed with PCR. A plasmid for disruption of the NSI (*slr0168*) was prepared similarly, but with a chloramphenicol resistance cassette. X*fpk* (phosphoketolase from *B. breve*, D6PAH1) was codon optimized and synthesized by Integrated DNA Technologies and overexpressed in the NSI under the control of *P*_*trc*_. The native genes encoding PhaA (*slr1993*) and PhaB (*slr1994*) were PCR amplified from the genome of *Synechocystis* and overexpressed in the NSI in the same way. Full segregation was verified with colony PCR.

All subcloning was performed in *E. coli* TOP10 or XL1-Blue, and plasmids were purified with miniprep before *Synechocystis* transformation. *Synechocystis* was typically transformed with 10–100 ng of replicating plasmid by electroporation [41], or by natural transformation in the case of genomic integration plasmids, and grown photoautotrophically on BG-11 agar plates.

### RT-qPCR

Total RNA was isolated from 5 mL of mid-log (OD_730_ = 1) cell cultures. RNA isolation and RT-qPCR was executed as previously described [42]. Levels of total RNA were used as reference for normalization.

### Western blotting

Expression of PhaJ, Ter, AdhE2, PhaA, PhaB and Xfpk was analyzed through Western blotting. 14–16 mL cell culture was centrifuged and resuspended in CelLytic B Cell Lysis Reagent (Sigma-Aldrich) supplemented with 1 mM PMSF. Acid-washed glass beads, 425–600 μm (Sigma-Aldrich), were added and the cells were lysed by vortexing for 20 min at 4 °C, followed by centrifugation for 4 min at 13,000×*g*, 4 °C. Supernatant containing 6–15 μg protein, determined with the bicinchoninic acid assay, was used for the Western blots. C-Myc-tagged PhaJ was detected with an anti-c-Myc mouse IgG (Invitrogen), FLAG-tagged Ter was detected with an anti-FLAG M2 mouse IgG (Sigma-Aldrich), Strep II-tagged AdhE2 was detected with an anti-Strep tag II mouse IgG (Qiagen) and 6His-tagged Xfpk with anti-His mouse IgG (Genscript). Polyclonal antibodies towards PhaA and PhaB were generated through rabbit immunizations.

### Metabolite quantification

*n*-butanol was quantified from the culture medium. 330 μL of cell culture was pelleted for 3 min at 16,000×*g* and the supernatant was collected. Isobutanol (5 μL of 0.1 %) was added to 300 μL supernatant as an internal standard. The supernatant was mixed vigorously with 100 μL (1/3 volume) dichloromethane for 30 s. The organic phase was analyzed via GC-FID (Hewlett Packard HP5890 Series II, or Shimadzu GC-2010 Plus). A CP–Chirasil-DEX CB column (25 m × 0.32 mm ID, 0.25 μm film thickness, Varian Chrompack) or a Stabilwax column (30 m × 0.25 mm ID, 0.25 μm film thickness, RESTEK) was used on the Hewlett Packard and Shimadzu instruments, respectively.

Glycogen was quantified from biological triplicates. Cell pellets from 1 mL cultures at OD_730_ 1 were washed in ddH_2_O and stored in −20 °C until further processing. Glycogen was isolated from the pellets as previously described [43]. Glycogen pellets were resuspended in 200 μL ddH_2_O and mixed with 10 μL of 54 % H_2_SO_4_ prior to incubation at 100 °C for 1 h. The samples were neutralized with NaOH and glycogen was quantified according to the protocol of Schlebusch et al. [10]. Glycogen from rabbit liver (Sigma-Aldrich) was used as standard.

Acetate was quantified from the culture medium with an Acetic Acid Assay Kit (K-ACET, Megazyme) following the manufacturer’s instructions. Samples were prepared from biological duplicates.

Acetyl-CoA levels were quantified from biological duplicates of 50 mL cultures at OD_730_ 1. The cultures were centrifuged for totally 8 min at 4 °C, 10,000×*g*, pellets were flash frozen in liquid nitrogen and stored at −80 °C until further processing. Pellets were resuspended in 200 μL of 1 M perchloric acid, acid-washed beads were added and samples were lysed by vortexing at 4 °C for 30 min. Lysates were centrifuged for 10 min at 13,000×*g*, 4 °C, supernatants were neutralized with 3 M KHCO_3_ followed by centrifugation for 2 additional min. The supernatants were run through 10 kDa cutoff centrifugal filters (Amicon Ultra-0.5 mL, Merck Millipore). Acetyl-CoA was quantified with an Acetyl-Coenzyme A Assay Kit (Sigma-Aldrich) according to manufacturer’s instructions.

NAD^+^ and NADH were quantified from 20 mL of culture, using an NAD^+^/NADH quantification colorimetric kit (BioVision). Cells were centrifuged for 10 min at 4500×*g*, washed in cold PBS and resuspended in 200 μL extraction buffer enclosed with the kit, supplemented with 50 U of Benzonase^®^ Nuclease (Sigma-Aldrich). Approximately 100 μL acid-washed glass beads were added to the samples before lysis on a vortexer for 30 min at 4 °C. Lysed cultures were centrifuged at 20,000×*g*, 4 °C for 5 min. The supernatant was run through a 10 kDa cutoff centrifugal filter (Sigma-Aldrich) for 45 min at 14,000×*g*, 4 °C. All samples were prepared from biological duplicates and measured according to manufacturer’s protocol in a CLARIOstar plate reader (BMG LABTECH). The results were normalized to protein content.

PHB was extracted from 30 mL of nitrogen replete cultures at OD_730_ 1, corresponding to 6.4 mg of dry cells, and 2.3 mg of DCW from nitrogen deplete cultures (see Additional file 1: Fig. S1 for OD_730_ to DCW conversions). All cultures were prepared in biological triplicates. The extractions were prepared as previously described [17, 44] and analyzed with RP-HPLC (1200 series, Agilent) using an Aminex HPX-87H column (300 × 7.8 mm, Bio-Rad Laboratories).

### Flux balance analysis of *Synechocystis* PCC 6803

The *Synechocystis* sp. PCC 6803 model of Knoop et al. [33] was used to perform flux balance analysis. The reconstructed network incorporates 677 genes, 759 reactions and 601 metabolites. The fermentative *n*-butanol pathway and two phosphoketolase reactions were added to the model. All simulations were performed using the COBRA toolbox 2.0 on Matlab [45]. Autotrophic conditions were simulated as “light limited” where photon flux was constrained to 18.7 mmol/gDCW/h and HCO_3_^−^ and CO_2_ uptake was unconstrained.

## Additional file

10.1186/s12934-015-0355-9 *Synechocystis* strains used in this study.
