# Supplementary material for: Genetic and nutrient modulation of acetyl-CoA levels in Synechocystis for n-butanol production
Source: Microb Cell Fact. 2015 Oct 16;14:167. doi: 10.1186/s12934-015-0355-9 (PMC4609045; doi:10.1186/s12934-015-0355-9)
Supplement: Supplementary file 1 — 10.1186/s12934-015-0355-9 Synechocystis strains used in this study. [file 12934_2015_355_MOESM1_ESM.docx]

Supplementary material

**Genetic and nutrient modulation of acetyl-CoA levels in *Synechocystis* sp. PCC 6803 for *n*-butanol production**.

Josefine Anfelt^a^, Danuta Kaczmarzyk^a^, Kiyan Shabestary^a^, Björn Renberg^a^, Johan Rockberg^a^, Mathias Uhlén ^a,b^, Jens Nielsen ^b,c^ and Elton P. Hudson^a†^

^a^School of Biotechnology, KTH - Royal Institute of Technology, Science for Life Laboratory, Stockholm, Sweden

^b^Novo Nordisk Foundation Center for Biosustainability, Technical University of Denmark, Hørsholm, Denmark

^c^Department of Chemical and Biological Engineering, Chalmers Institute of Technology, Gothenburg, Sweden

Promoters used in this study, with the RBS (Biobrick BBa_B0034 in the case of *P_trc_*) underlined:

***P_trc_*:** ATGAGCTGTTGACAATTAATCATCCGGCTCGTATAATGTGTGGAATTGTGAGCGGATAACAATTTCACACAAAAGAGGAGAAA

***P_psbA2_*:** AACTGACTGACCACTGACCTTAAGAGTAATGGCGTGCAAGGCCCAGTGATCAATTTCATTATTTTTCATTATTTCATCTCCATTGTCCCTGAAAATCAGTTGTGTCGCCCCTCTACACAGCCCAGAACTATGGTAAAGGCGCACGAAAAACCGCCAGGTAAACTCTTCTCAACCCCCAAAACGCCCTCTGTTTACCCATGGAAAAAACGACAATTACAAGAAAGTAAAACTTATGTCATCTATAAGCTTCGTGTATATTAACTTCCTGTTACAAAGCTTTACAAAACTCTCATTAATCCTTTAGACTAAGTTTAGTCAGTTCCAATCTGAACATCGACAAATACATAAGGAATTAT

**Fig. S1.** Correlation between OD_730_ and dry cell weight (DCW) of wild-type *Synechocystis* at nitrogen replete (N+) and deplete (N-) conditions.

**Fig. S2.** A) Gene transcription changes during nitrogen starvation of strain JA02. Measurements were with RT-qPCR at nitrogen replete (N+) and 72 h of nitrogen deplete (N-) conditions. Nitrogen starvation was accompanied by a 8-9-fold induction of *phaA* and *phaB*, whereas the transcription of the heterologous genes changed less than 2-fold. B) Western blot of Ter from strain JA04 cultured under extended (3, 7 and 12 days) nitrogen starvation (N-) or nitrogen replete (N+) conditions. A high expression level was detected even after 12 days of starvation.

**Fig. S3.** A) Butanol titers after 14 days of growth. B) Butanol yields from strain JA02 cultured for seven days in BG-11 medium supplemented with varying nitrate concentrations. Butanol production strongly depends on the nitrate content in the growth medium. For media composition see Materials and methods. C) PHB accumulation over time in wild-type at nitrogen replete (N+), deplete (N-) and phosphorous deplete (P-) conditions. As nutrient concentrations in the growth media decrease with time, PHB starts to accumulate also in the nitrate-supplemented cultures.

**Table S1**. Quantified metabolites at nitrogen replete (N+) and deplete (N-) conditions. Glycogen levels are relative to wild-type at N-, which is defined as 100 %.

ND: not detectable.

|  | WT | | JA02 | | JA04 | | JA05 | | JA06 | |
| --- | --- | --- | --- | --- | --- | --- | --- | --- | --- | --- |
|  | N+ | N- | N+ | N- | N+ | N- | N+ | N- | N+ | N- |
| Glycogen (Rel. %) | 3.5 ± 0.2 | 100 ± 6 | 4.3 ± 0 | 103 ± 3 |  |  |  |  |  |  |
| Acetyl-CoA/DCW (μg/g) | 19 ± 5 | 34 ± 6 |  |  | 9 ± 0.3 | 29 ± 4 |  |  | 113 ± 10 | 43 ± 4 |
| Acetate (mg/L) | ND | ND |  |  | 6.4 ± 0.5 | 1.8 ± 0.5 | 14 ± 0.5 | 13 ± 0.8 | 19 ± 0.3 | ND |
| NAD^+^/NADH | 5.5 ± 1.4 | 34 ± 16 | 4.1 ± 0.3 | 9.2 ± 3.2 |  |  |  |  |  |  |
| PHB/DCW (%) | ND | 47 ± 1 |  |  |  |  |  |  |  |  |

**Fig. S4.** Western blot detection of butanol pathway enzymes in cell lysates from strain JA03, JA04 and JA05 at nitrogen replete (N+) and deplete (N-) conditions.

A) Detection of PhaA and PhaB from 15 μg (N+) or 6 μg (N-) of total protein. PhaA and PhaB were significantly upregulated in strain JA03 and JA05 containing an additional *phaAB* cassette in the NSI. B) Detection of exogenous butanol enzymes from 6 μg of total protein per sample. Expression levels of PhaJ, Ter and AdhE2 were similar between strains with (JA05) and without (JA04) PhaA and PhaB overexpression.

**

**Fig. S5.** Acetyl-CoA concentrations in wild-type *Synechocystis* PCC 6803 and *Synechococcus* PCC 7942­ at nitrogen replete (N+) conditions.

**Fig. S6.** Western blot detection of N-terminally 6His-tagged Xfpk in strain JA07 at nitrogen replete (N+) and deplete (N-) conditions. 12 μg of total protein was loaded from each sample.

­­

**Table S2.** Additional *Synechocystis* strains used in this study.

| **Strain** | **Plasmid** | **Genome modification** |
| --- | --- | --- |
| JA08 | pJA8-*P_trc_ phaJ ter pduP* | Δ*phaEC*::SpR |
| JA09 | pJA8-*P_trc_ phaJ ter pduP yqhD* | Δ*phaEC*::SpR |
| JA13 | pJA8-*P_trc_ phaJ ter* | Δ*phaEC*::SpR |

**Fig. S7.** A) Specific titers of butanol after 8 days of growth at nitrogen replete (N+) and deplete (N-) conditions. B) Expression levels of butanol pathway enzymes in strain JA09 compared to JA04 at nitrogen replete conditions, detected with Western blot. 12 μg of total protein in cell lysates was loaded from each sample.
